# Supplementary figures and images for: Prevalence and genotype-specific distribution of human papillomavirus in Burundi according to HIV status and urban or rural residence and its implications for control
Source: PLoS One. 2019 Jun 25;14(6):e0209303. doi: 10.1371/journal.pone.0209303 (PMC6592514; doi:10.1371/journal.pone.0209303)

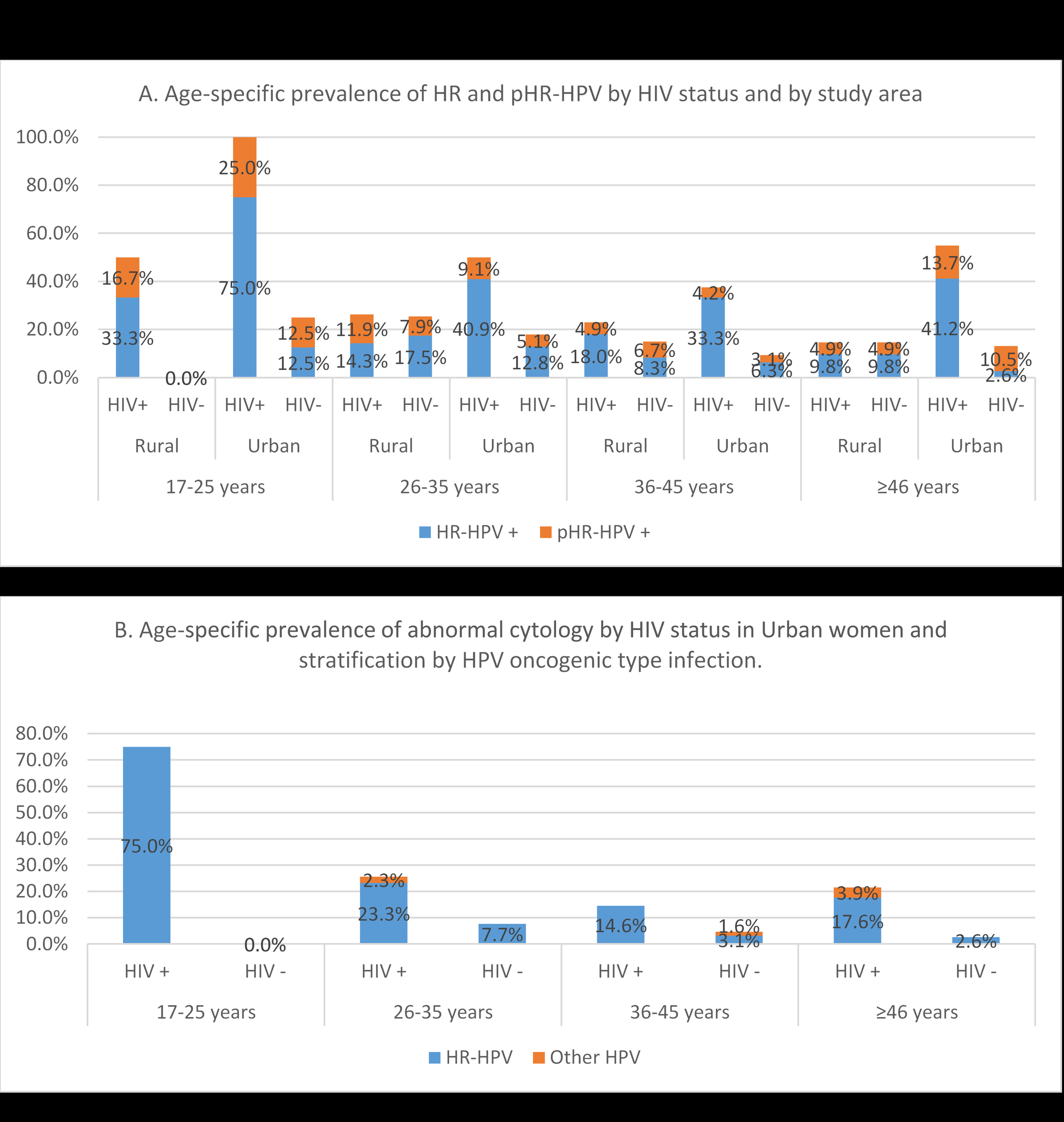

Supplement: S1 Fig — Age-specific prevalence of HPV (A) and of abnormal cytological results (B) stratified by HIV status in rural and urban women, Burundi. (TIF) [file pone.0209303.s001.tif]

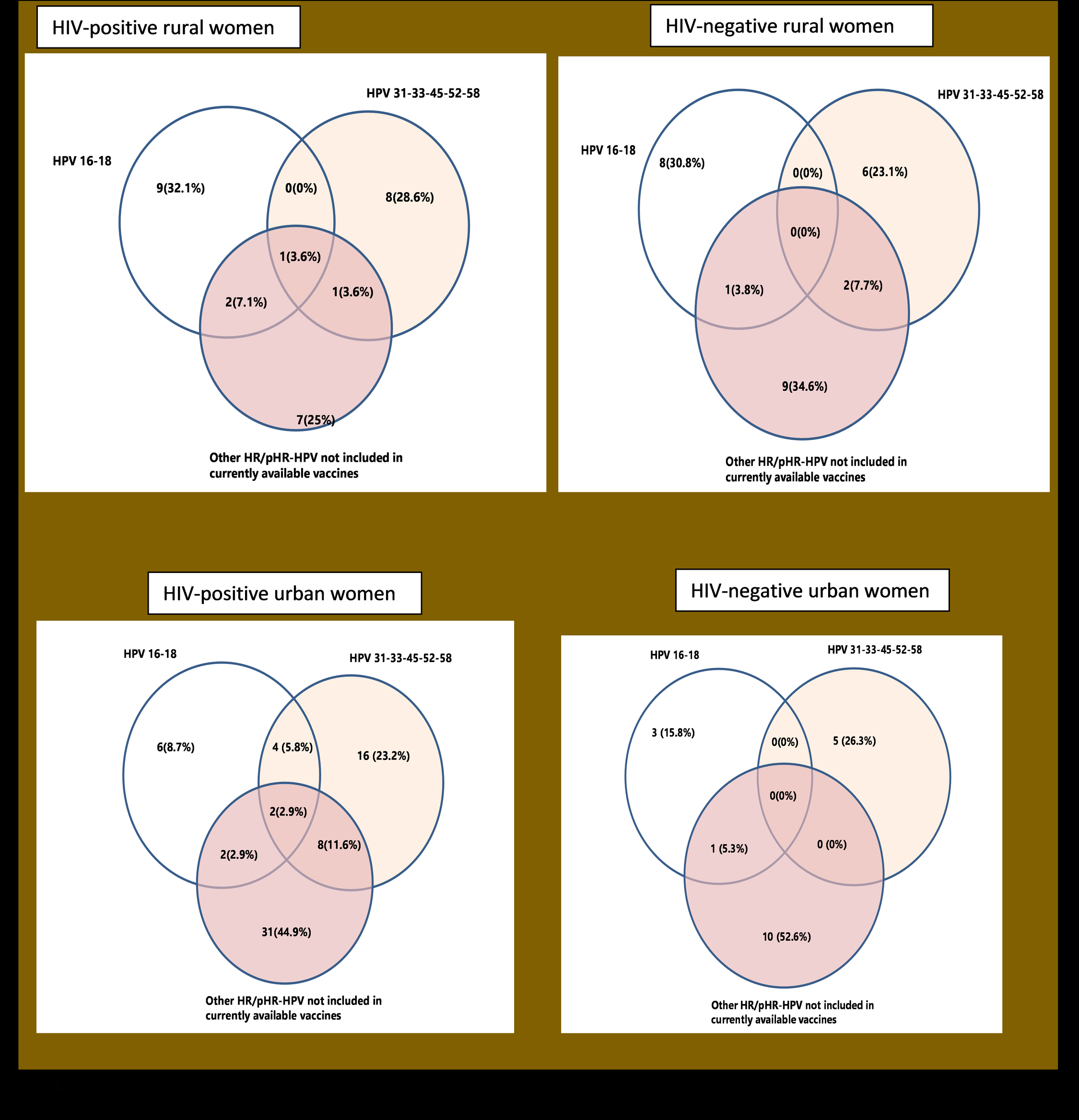

Supplement: S2 Fig — (TIF) [file pone.0209303.s002.tif]
